# A Novel Elucidation for Synflorescences of Chinese Bamboos

**DOI:** 10.3390/plants13010029

**Published:** 2023-12-21

**Authors:** Zhuo-Yu Cai, Nian-He Xia

**Affiliations:** 1 State Key Laboratory of Plant Diversity and Specialty Crops, South China Botanical Garden, Chinese Academy of Sciences, Guangzhou 510650, China; caizhuoyu@scbg.ac.cn; 2 Guangdong Provincial Key Laboratory of Digital Botanical Garden, South China Botanical Garden, Chinese Academy of Sciences, Guangzhou 510650, China; 3 South China National Botanical Garden, Guangzhou 510650, China; 4 University of Chinese Academy of Sciences, Beijing 100049, China

**Keywords:** morphology, basic flowering branch, pseudospikelet, Bambusoideae

## Abstract

The objective of this work is to elucidate the flowering structures of Chinese bamboos applying the synflorescence concept. To keep in line with grasses, the bamboo synflorescence is defined as a whole culm or a whole branch terminating in an inflorescence. For the first time, the repetitive and fundamental unit of bamboo synflorescences is clearly identified and termed as the “basic flowering branch”. The basic flowering branch could be considered as the most simplified synflorescence for a bamboo species. Applying the synflorescence concept, the pseudospikelet is interpreted as a sort of basic flowering branch rather than a spikelet. Consequently, the synflorescence development pattern is consistent throughout the whole family. This study also marks the first recognition of both pseudospikelets and true spikelet flowering branches within the same bamboo synflorescence, which is observed in the genera *Brachystachyum*, *Semiarundinaria* and *Menstruocalamus*.

## 1. Introduction

For a very long period, there was no recognized circumscription for the bamboo inflorescence. Roxburgh described the whole flowering bamboo plant as an entire and immense panicle [1,2]. In 1868, Munro, referring to the opinion of Nees von Esenbeck [3], defined it as a panicle, sometimes reducing to a raceme or spike [4]. In 1896, Gamble considered that bamboo inflorescences are various, usually a large compound panicle with spicate branches [5]. In 1913, Camus determined bamboo inflorescences as a more or less ramose panicle formed by spikes, sometimes in a contracted panicle or in a spike-shaped raceme [6]. In 1966, McClure summarized the bamboo inflorescence as an axis or a system of axes emanating from a common axis, the primary rachis, which ends in a spikelet and so does every branch of every order [7]. Additionally, he distinguished them into two forms: the determinate/semelauctant (once grown) inflorescence consisting of true spikelets and the indeterminate/iterauctant (repeatedly grown) inflorescence consisting of pseudospikelets [7,8]. The term “pseudospikelet”, coined by McClure, refers to the special reproductive structure of some bamboos, which is a branch with a superficial spikelet appearance and basally clothed by gemmiferous bracts and a prophyll [7,9]. Each gemmiferous bract subtends a bud instead of a flower, which will grow into a secondary pseudospikelet [7,9]. McClure’s perspective has gained widespread acceptance among scholars [10,11,12,13,14,15,16,17,18]. However, adopting his perspective, the inflorescence comprised of pseudospikelets became very peculiar in the family, especially regarding the development pattern [19].

The conventional terminology used for analyzing and describing the inflorescence in Poaceae Barnhart (including both grasses and bamboos) is inaccurate [19]. To reach a better understanding, the concept of synflorescence, introduced by Troll [20] and expounded by Weberling [21], has been employed to analyze and describe flowering structures not only in Poaceae (grasses) [22,23,24,25,26,27] but also in many other angiosperm families such as Amaranthaceae Juss., Aristolochiaceae Juss., Asteraceae Bercht. & J. Presl, Commelinaceae Mirb., Cyperaceae Juss., Eriocaulonaceae Martinov, Euphorbiaceae Juss., Lowiaceae Ridl., Rhamnaceae Juss. etc. [28,29,30,31,32,33,34,35].

Some scholars have contributed to applying the synflorescence concept for the flowering structures of both herbaceous bamboos [36] and woody bamboos [13,16,37,38,39]. However, the synflorescence concept is comprehended by grass experts and bamboo experts differently. The synflorescence recognized by some bamboo experts [16] corresponds to the inflorescence (a part of the synflorescence) identified by grass experts [22,23]. The discrepancy between the two interpretations would cause unnecessary confusion. On the other hand, bamboos have diverse and intricate patterns of branching habit in the vegetative apparatus, as well as in the reproductive parts, which complicate its analysis [40]. In the past, some Chinese scholars attempted to identify a repetitive and fundamental unit from the complex branching system and used it as a research object when they studied the reproductive characteristics of some woody bamboos [41,42]. Although they only provided an ambiguous concept of this unit, named the “ultimate flowering branch”, it enlightens a feasible approach to analyzing the intricate flowering structures of bamboos.

In China, there are approximately 37 bamboo genera, exhibiting a significant morphological diversity in bamboo synflorescences [17,43,44,45,46,47]. Therefore, bamboos from China (also containing some material from neighboring areas) are selected as the research objects for the following aims: (1) to discuss and confirm the delimitation of synflorescence in woody bamboos, (2) to find a practicable method to analyze and describe the bamboo flowering structures by applying the synflorescence concept, (3) to re-interpret the pseudospikelet of woody bamboos, and (4) to elucidate the synflorescence for some bamboos.

## 2. Results and Discussion

### 2.1. The Synflorescence Concept of Woody Bamboos

In Bambusoideae Luerss., Calderón and Soderstrom employed the concept of synflorescence to analyze three herbaceous bamboo genera for the first time [36]. The synflorescence they adopted is the whole floral aggregation in a plant, viz., the system of the main florescence with its coflorescences [36]. Later, Soderstrom and Londoño interpreted the flowering structures of *Alvimia* Calderón ex Soderstrom & Londoño, a woody bamboo, with the synflorescence concept that was previously applied to herbaceous bamboos [37]. Following the interpretation of Calderón and Soderstrom, Young and Judd, and Wong regarded the pseudospikelet clusters as discrete synflorescences [13,39]. But with this interpretation, Young and Judd mentioned that the length of branches and distance between internodes varies in each complement of branches, and it is often difficult to delimit a “cluster” in a non-arbitrary fashion [39]. In other words, it would be difficult to recognize the main florescence when the flowering structures present a complicated axis ramification, which is very common in woody bamboos. Stapleton discussed the synflorescence for both grasses and bamboos and suggested that such an interpretation aligns grass “inflorescences” with those of other families, although more research is needed to examine the advantages of the new terminology [38]. However, he did not seem to provide a definition for bamboo synflorescences. Judziewicz et al. defined the synflorescence of bamboos as the flowering structure that emerges from the apex of a leafy branch, as that branch usually grows from a major axis (or the culm apex) [16]. The synflorescence defined by Judziewicz et al. is equivalent to inflorescence or the unit of inflorescence that is adopted by the grass experts [16,19,26]. Reinheimer and Vegetti considered a synflorescence as a shoot system of the plant originated from the apical meristem or axillary buds [23]. In other words, each shoot is a synflorescence [22].

**Figure 1:**
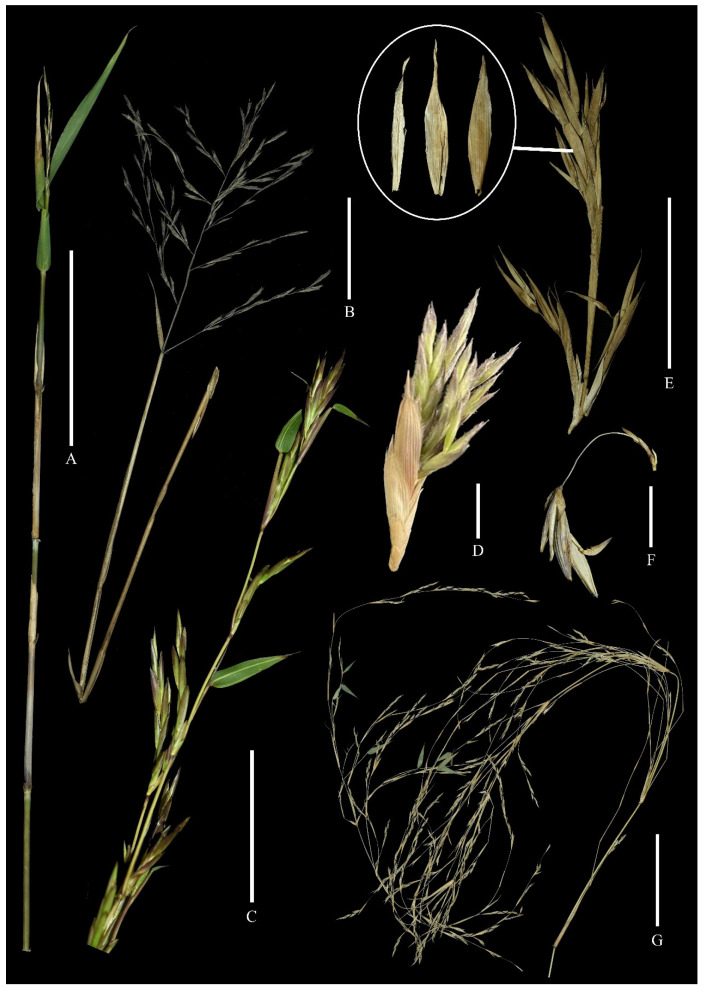
Flowering branches of woody bamboos. (**A**) A bamboo shoot of *Indocalamus longiauritus* terminating in an immature inflorescence. (**B**) A flowering branch of *Indocalamus herklotsii*. (**C**) Flowering branches of *Bambusa albolineata*. (**D**) A flowering branch cluster of *Brachystachyum densiflorum*. (**E**) Flowering branches of *Semiarundinaria fastuosa*, images in the circle showing the morphology transition of the lowermost glume from the proximal spikelet to the distal spikelet (from left to right). (**F**) A short flowering branch of *Petrocalamus luodianensis*. (**G**) Flowering branches of *Neomicrocalamus* sp., showing the complex branching system. Scale bars: (**A**,**G**) (10 cm); (**B**,**C**,**E**) (5 cm); (**D,F**) (1 cm).

**Figure 2:**
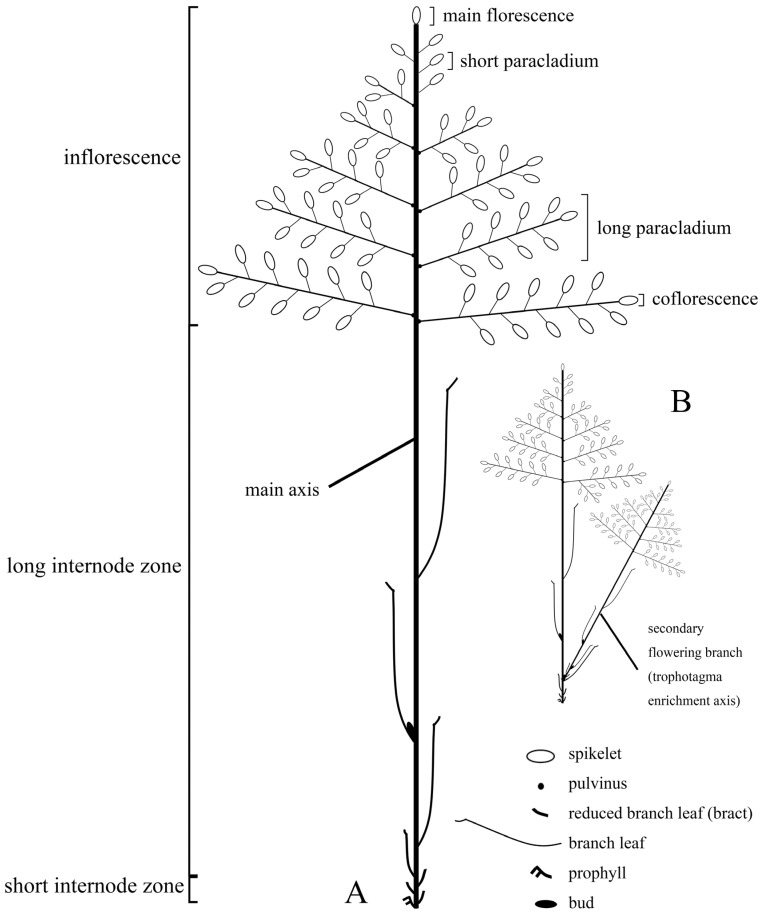
Diagram of flowering branches of *Gelidocalamus fengkaiensis*. (**A**) A basic flowering branch. (**B**) A synflorescence comprised of two flowering branches.

**Figure 3:**
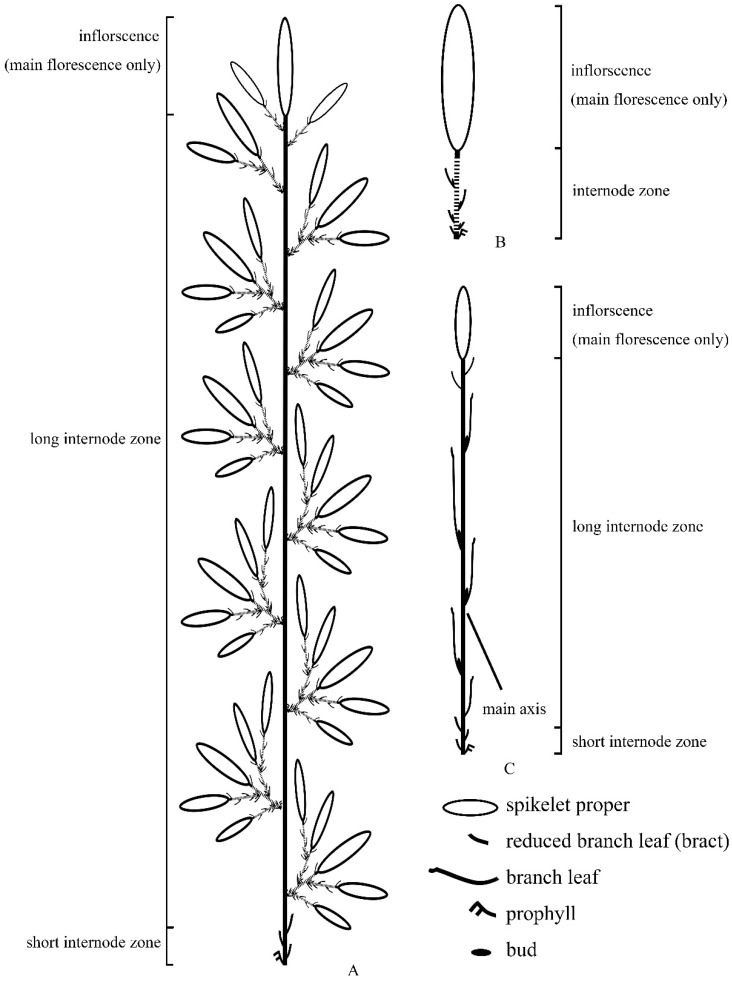
Diagram of flowering branches of *Bambusa contracta*. (**A**) A synflorescence comprised of long flowering branches and short flowering branches. (**B**) A short basic flowering branch (lateral pseudospikelet). (**C**) A long basic flowering branch (terminal pseudospikelet). Dotted lines denote stretched short axes for diagram.

Given that bamboos belong to Poaceae, the synflorescence of bamboos should be reconciled with that of grasses. In our view, a bamboo synflorescence should encompass a whole culm terminating in flowering structures, along with all branches born on the culm (Figure 1A, Figure 2B and Figure 3A), or only include a whole branch terminating in flowering structures, along with all branches born on the branch, when the main culm apex remains vegetative. Because of the complex branching system of woody bamboos (Figure 1G) and variable interpretations of actual synflorescence structures, it is admittedly difficult to analyze and describe a bamboo synflorescence. Therefore, in order to avoid confusion and problems in interpretation, we propose that the basic flowering branch, which is analyzable and recognizable in all zones of a synflorescence, should be chosen as the research object for bamboo synflorescence studies.

### 2.2. The Basic Flowering Branch

We refined the concept of previously known as “ultimate flowering branch” and renamed it the “basic flowering branch”. The basic flowering branch is a repetitive and fundamental unit in bamboo synflorescences. It is defined as a single flowering branch that begins from the basal prophyll, embracing it, and ends with the terminal spikelet (Figure 2A). The basic flowering branch excludes any secondary or higher-order branch growing on it. It could be recognized as a most simplified synflorescence for a bamboo species. Usually, several to many basic flowering branches constitute a synflorescence. According to the synflorescence concept, some structures could also be identified within the basic flowering branch, such as the main axis, the inflorescence, the long internode zone and short internodes zone, and so on. Further details are elucidated below.

**The main axis.** The axis bearing all appendages on the basic flowering branch is referred to as the main axis (Figure 2). From the distal end to the proximal end, the main axis can be divided into three parts: the inflorescence, the long internode zone and the short internodes zone.

**The inflorescence.** The term “inflorescence” we used here is equivalent to “the unit of the inflorescence” (the UIF) [22]. In most grasses, the inflorescence is easily identified as the product of the shoot apical meristem after the transition to flowering, and it is a discrete structure that is terminal on the culm [19]. The inflorescence of woody bamboos, which possess true spikelets, is similar to that of grasses and can be easily identified [19] (Figure 1A,B and Figure 2). The spikelet on the main axis apex is characterized as the main florescence and the other spikelets, in the inflorescence, are the coflorescences [26]. In the inflorescence, the number of spikelets varies from one to many, depending on different taxa. The paracladium refers to the axis bearing coflorescences which can be distinguished into two types: the short paracladium and the long paracladium (Figure 2A). The short paracladium reduces to a single terminal spikelet, while the long paracladium terminates in a spikelet and could ramify second-order paracladia, which also terminate in spikelets [27]. Paracladia are bare and not covered by either “bracts” or prophylls. Sometimes, paracladia are basally covered by a scale which is commonly small and membranous. Some of these scales are enlarged into leather sheathes in certain genera, such as *Fargesia* Franch [48].

**The long internode zone and the short internode zone.** On the main axis, two internode zones exist beneath the inflorescence, the long internode zone and the short internode zone, which are formed by distantly spaced distal nodes and closely spaced basal nodes, respectively [49] (Figure 2A). Each distantly spaced node is initially subtended by either a branch leaf with rudimentary blades or a foliage leaf with fully developed blades. The leaf structure subtending each closely spaced node takes on a bract-like appearance. The most basal part of the short internode zone, which is also the most basal part of the whole basic flowering branch, is embraced by one or two prophylls. The prophyll is the first phyllome of the flowering branch. Each node, whether in the long or short internode zone, probably bears a prophyllate bud, which is able to develop into a secondary branch. This kind of development could repeat on each order of branches. For a synflorescence, it includes the mother flowering branch and all orders of branch (namely trophotagma enrichment axes, see below) born on its long internode zone. Branches generated on the short internode zone would be recognized as a separated new synflorescence [49,50].

**The trophotagma enrichment axis.** In the grass synflorescence, besides the paracladium in the inflorescence, a second type of axis occurs on the long internode zone, which is known as the trophotagma enrichment axis/paraclade of the trophotagma zone/long paracladia with the trophotagma. The trophotagma means the feeding (trophe) part (tagma; plural: tagmata) referring to leaf structures, viz., prophylls, bracts, foliage leaves, and so on [27,51]. The trophotagma enrichment axis is a shoot with leaf structures and ends with an entire inflorescence [22]. We agree with the opinion that the trophotagma enrichment axis must be differentiated from the paracladium in the inflorescence which lacks trophotagma [22,26,27]. In different grass taxa, the trophotagma enrichment axis has variable branching degrees, ranging from one to several orders [22,52].

For woody bamboos, the trophotagma enrichment axis is morphologically similar to its mother flowering branch, and the trophotagma enrichment axes commonly exhibit a high branching degree, contributing to the complex axis system of bamboo synflorescences. Due to the complexity of the axis system, identifying the mother flowering branch and the trophotagma enrichment axis becomes challenging. To address this issue, we propose treating mother flowering branches and trophotagma enrichment axes as the same repetitive unit (namely the basic flowering branch) of synflorescences, as they share similar structures (Figure 2B).

### 2.3. The Development Pattern and the Pseudospikelet

McClure proposed two kinds of development patterns for bamboo “inflorescences” [7,8]. The semelauctant/determinate “inflorescence” would complete its growth in a single grand period [7,8]. This development is associated with the true spikelet. In contrast, pseudospikelets would continuously produce higher-order pseudospikelets from their basal axillary buds. Hence, McClure interpreted a pseudospikelet cluster as an iterauctant/indeterminate “inflorescence” [7,8]. The unique development pattern makes the pseudospikelet distinctive in the family Poaceae, as all other members feature the semelauctant development [19].

Pseudospikelets are spikelet-like branches with upper parts having some aspects of spikelet identity and lower parts having some aspects of branch identity [7,9,19]. Applying the synflorescence concept, the pseudospikelet is better to be recognized as a sort of basic flowering branch rather than a spikelet. A pseudospikelet is a concise flowering branch. The upper part of pseudospikelets is the spikelet proper, which is properly identified as a simplified inflorescence reducing to a single spikelet, namely the main florescence (Figure 3B). The lower part of pseudospikelets is the basal axis, which is clothed by bract-like branch leaves with buds in their axils and prophylls. This part is commonly too short to clearly discriminate the long internode zones and the short internode zones (Figure 3B). Those buds can grow out into secondary pseudospikelets that closely resemble the primary one (Figure 3A). There are two kinds of primary pseudospikelets recognized by McClure: “lateral ones are sessile; a terminal one is made pedicellate by the distal internode…” [7]. Thus, bamboos, featuring pseudospikelets, have two kinds of basic flowering branches: the terminal one (long axis, long basic flowering branch) (Figure 3C) and the lateral one (short axis, short basic flowering branch) (Figure 3B). The main axis of the long basic flowering branch is long enough to distinguish two internode zones.

In this study, the pseudospikelet is regarded as the flowering branch. Its inflorescence is restricted to the single terminal spikelet which clearly exhibits a semelauctant nature. In other words, the inflorescence of all bamboos, like grasses, is consistently semelauctant. The repeating development of pseudospikelets is similar to the way in which true spikelet flowering branches produce secondary branches. The iterauctant development is actually an attribute of the flowering branch or the synflorescence. However, for some taxa, their synflorescences do not produce secondary branches. In this interpretation, the development of both inflorescence and synflorescence is consistent throughout the whole family.

### 2.4. The Characters of Some Bamboo Synflorescences

Some flowering branch characteristics were summarized for all the material that we examined (Table 1). The identification of flowering branch types is provisional. Intermediate types between the true spikelet flowering branch and the pseudospikelet are necessarily to be recognized in future studies. Some cases are explained here.

**The leafless flowering branch.** The majority of the bamboos we studied could generate inflorescences at both the apex of leafy branch and the leafless branch. However, it has not been observed that flowering structures emerging from the apex of leaf branches in bamboos with a single ultimate foliage leaf, such as *Gelidocalamus fengkaiensis* N.H. Xia & Z.Y. Cai, *Indosasa shibataeoides* McClure and *Shibataea* sp. During a flowering episode of *G. fengkaiensis*, the apical meristem of a foliage leaf branch, tightly rolled by a very thickened leaf sheath, is dormant or even eventually senescent [53].

**The synflorescence of *Bonia*.** *Bonia* Balansa is charactered by pseudospikelets [54,55]. Hence, it possesses two kinds of basic flowering branches. Based on our observation, secondary short basic flowering branches of *Bonia* spp. typically develop from buds in the prophyll axils rather than in the axils of bract-like branch leaves. Thus, a pseudospikelet cluster of *Bonia* is not a multi-branched synflorescence. Instead, it is an aggregation of many unbranched synflorescences, as branches generating from the short internode zone would be considered as separated synflorescences.

**The synflorescence of *Petrocalamus*.** The synflorescence of *Petrocalamus* Z.P. Wang, N.X. Ma & W.Y. Zhang features a large number of short basic flowering branches which bear some resemblance to pseudospikelets. The differences include the potentially branched inflorescences (when branched, the inflorescence bears more than one spikelet) and the slender axis that separates the spikelets from the basal axis (Figure 1F). For typical pseudospikelets, the inflorescence is always unbranched, containing only one spikelet, and the spikelet is adjacent to the basal axis. Furthermore, the short basic flowering branch of *Petrocalamus* is incapable of producing secondary flowering branches, while typical pseudospikelets can vigorously produce several orders of pseudospikelets.

**Figure 4:**
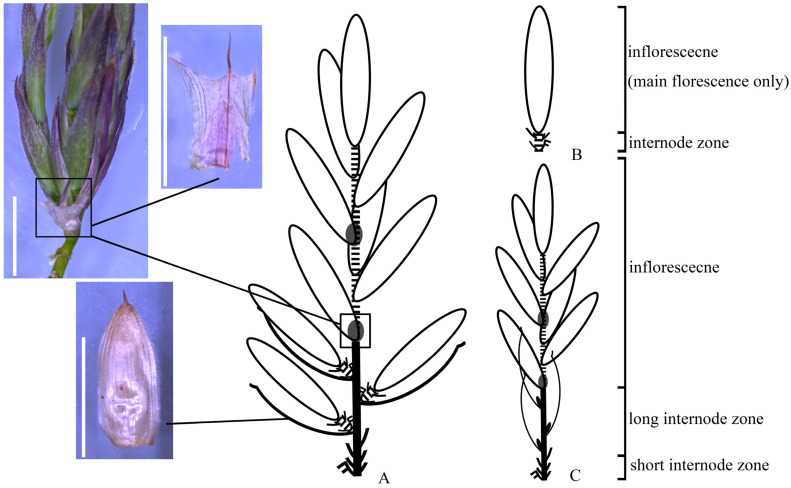
Diagram of flowering branches of *Brachystachyum densiflorum*. (**A**) A synflorescence comprised of long flowering branches and short flowering branches. (**B**) A short basic flowering branch (pseudospikelet). (**C**) A long basic flowering branch with true spikelets. Scale bars (white bars): 1 cm. Legends are the same as in Figure 3.

**The synflorescence of *Brachystachyum*.** A re-examination of the synflorescence of *Brachystachyum densiflorum* (Rendle) Keng, the generic type, is made here [56]. The “spikelets” of this species are conventionally recognized as pseudospikelets [57]. However, its synflorescence is more than just pseudospikelets. Two kinds of basic flowering branches are identified in it. The long basic flowering branch terminates in an inflorescence with several sessile true spikelets (Figure 4), as they lack prophylls and gemmiferous “bracts” (branch leaves). These true spikelets are basally embraced by a small membranous “sheath”, corresponding to the scale at the paracladium base of the inflorescence with true spikelets (Figure 4). The short basic flowering branch, born on the long internode zone of the long basic flowering branch, could be identified as the pseudospikelet, as it has a spikelet appearance; however, it is basally clothed by a prophyll and several gemmiferous “bracts” (Figure 4B). The distinctive synflorescence structures of *B. densiflorum* may well be attributed to its hybrid origin. Some studies have indicated that *B. densiflorum* originated from the natural hybridization between *Phyllostachys* Siebold & Zucc. species (maternal) and Chinese *Pleioblastus* Nakai species (paternal). The former possesses pseudospikelets, and the latter possesses true spikelet flowering branches [58,59].

**The synflorescence of *Semiarundinaria*.** The hybrid origin of *Semiarundinaria* Makino ex Nakai also has been indicated by some molecular evidence with the maternal parent from *Pleioblastus* and the paternal parent from *Phyllostachys* [58]. The synflorescence of this genus is similar to that of *Brachystachyum*. The short basic flowering branch somehow fits the pseudospikelet definition. The inflorescence of the long basic flowering branch is characterized by several true spikelets. The lowermost leaf structure of spikelets on the inflorescence of the long basic flowering branch changes consecutively from the base to the apex. The lowermost leaf structure of the lowermost spikelet is veined and conspicuously two-keeled, while that of the upper ones are non-keeled and more glume-like (Figure 1E). The lowermost leaf structure is more reasonably recognized as the glume; therefore, these spikelets are identified as true spikelets.

**The synflorescence of *Menstruocalamus*.** The taxonomy of this monotypic genus, *Menstruocalamus* T.P. Yi, is controversial. The generic type, *Menstruocalamus sichuanensis* (T.P. Yi) T.P. Yi, was initially placed in *Sinobambusa* Makino ex Nakai by Yi, due to its pseudospikelets and three stamens [60]. Then, it was transferred to *Chimonobambusa* by Wen [61]. However, Yi re-identified its “spikelets” as true spikelets and transferred it to the new genus *Menstruocalamus* [62]. In fact, the synflorescence of *Menstruocalamus* is also similar as *Brachystachyum*, featuring both the true spikelet flowering branch and the pseudospikelet. Moreover, *Menstruocalamus* also originated from hybridization, for which the putative parents are from *Chimonobambusa* (pseudospikelets) and *Bashania* Keng f. & T.P. Yi (true spikelet flowering branches) [59,63].

## 3. Materials and Methods

Studies were performed in species (Table 2). Specimens from the following herbaria were examined: IBSC, NF, PE, SWFC and SYS. Herbarium acronyms follow the Index Herbariorum (https://sweetgum.nybg.org/science/ih/ (accessed on 26 October 2023)). Living samples, specimens, and literature are used for analyses. Flowering materials were dissected under a stereo microscope (MZ101, Mshot, Guangzhou, China). Images were taken with the camera attachment (MSX2, Mshot, Guangzhou, China). The terms applied to the flowering structures in the analysis and discussion mainly follow the synflorescence concept [22,23,24,26,38].

## 4. Conclusions

The bamboo synflorescence is better defined as a whole culm or a whole branch (encompassing all branches arising the culm or the branch) terminating in an inflorescence, which aligns the bamboo synflorescence with that of grasses. The basic flowering branch is now clearly identified and elucidated for the first time. It could be used as the research object avoiding the probable confusion caused by the complex axis system when studying bamboo synflorescences. The pseudospikelet is recognized as a kind of basic flowering branch. Consequently, the development pattern of synflorescences is reconciled for Poaceae. In this family, all inflorescences should be semelauctant and synflorescences could be iterauctant. Applying the concept of synflorescence allows for a more accurate interpretation and understanding of bamboo flowering structures. As such, more informative characters could be identified, which would be of benefit to not only the bamboo classification but also other research endeavors.

## Figures and Tables

**Table 1:** Flowering branch characteristics.

Species	Flowering Branch Type	Paracladium	Scale of Paracladium
**Bambuseae**			
*Bambusa albolineata*	P	absent	absent
*Bambusa contracta*	P	absent	absent
*Bambusa corniculata*	P	absent	absent
*Bambusa cornigera*	P	absent	absent
*Bambusa subtruncata*	P	absent	absent
*Bonia levigata*	P	absent	absent
*Bonia saxatilis*	P	absent	absent
*Dendrocalamus brandisii*	P	absent	absent
*Dendrocalamus farinosus*	P	absent	absent
*Dendrocalamus* sp.	P	absent	absent
*Gigantochloa brachystachya*	P	absent	absent
*Gigantochloa maneensis*	P	absent	absent
*Melocanna humilis*	P	absent	absent
*Melocanna baccifera*	P	absent	absent
*Neololeba atra*	P	absent	absent
*Neomicrocalamus prainii*	P	absent	absent
*Neomicrocalamus* sp.	P	absent	absent
*Temochloa* sp.	P	absent	absent
*Schizostachyum dakrongense*	P	absent	absent
*Schizostachyum hainanense*	P	absent	absent
**Arundinarieae**			
*Acidosasa carinata*	T	present	small
*Acidosasa chinensis*	T	present	small
*Acidosasa gracilis*	T	present	small
*Ampelocalamus actinotrichus*	T	present	absent
*Brachystachyum densiflorum*	T + P	absent or present	absent or enlarged
*Chimonobambusa marmorea*	P	absent	absent
*Fargesia sapaensis*	T	present	small
*Fargesia spathacea*	T	present	enlarged
*Ferrocalamus rimosivaginus*	T	present	absent
*Ferrocalamus strictus*	T	present	absent
*Gelidocalamus fengkaiensis*	T	present	absent
*Indocalamus herklotsii*	T	present	absent
*Indocalamus longiauritus*	T	present	absent
*Indocalamus sinicus*	T	present	absent
*Indosasa hispida*	P	absent	absent
*Indosasa shibataeoides*	P	absent	absent
*Indosasa singulispicula*	P	absent	absent
*Khoonmengia honbaensis*	T	present	absent
*Menstruocalamus sichuanensis*	T + P	absent or present	absent or small
*Oligostachyum oedogonatum*	T	present	small
*Oligostachyum shiuyingianum*	T	present	small
*Oreocalamus Utilis*	P	absent	absent
*Petrocalamus luodianensis*	T	absent	absent
*Petrocalamus microphyllus*	T	absent or present	absent or small
*Phyllostachys bambusoides*	P	absent	absent
*Phyllostachys danxiashanensis*	P	absent	absent
*Pleioblastus* × *kongosanensis*	T	present	small
*Pleioblastus* sp.	T	present	small
*Pseudosasa cantorii*	T	present	small
*Pseudosasa palidiflora*	T	present	small
*Ravenochloa wilsonii*	T	present	absent
*Sasamorpha sinica*	T	present	absent
*Semiarundinaria fastuosa*	T + P	absent or present	absent or enlarged
*Shibataea* sp.	P	absent	absent
*Sinosasa longiligulata*	T	present	absent
*Yushania rugosa* aff.	T	present	absent
*Yushania* sp.	T	present	absent

“T” refers to the true spikelet flowering branch and “P” refers to the pseudospikelet.

**Table 2:** Species and material studied.

Species	Voucher
**Bambuseae**	
*Bambusa albolineata* L.C. Chia	Z.Y. Cai CZY193 (IBSC)
*Bambusa contracta* L.C. Chia & H.L. Fung	Q.M. Qin & J.B. Ni QQM39(IBSC)
*Bambusa corniculata* L.C. Chia & H.L. Fung	Q.M. Qin & J.B. Ni QQM40 (IBSC)
*Bambusa cornigera* McClure	Q.M. Qin & J.B. Ni QQM41 (IBSC)
*Bambusa subtruncata* L.C. Chia & H.L. Fung	Q.M. Qin & J.B. Ni QQM16 (IBSC)
*Bonia levigata* (L.C. Chia, H.L. Fung & Y.L. Yang) N.H. Xia	Z.Y. Cai & X.R. Zheng CZY24 (IBSC)
*Bonia saxatilis* (L.C. Chia, H.L. Fung & Y.L. Yang) N.H. Xia	Nan Zhu Di 5533 (IBSC)
*Dendrocalamus br&isii* (Munro) Kurz	J.R. Xue et al. 895 (SWFC)
*Dendrocalamus farinosus* (Keng & Keng f.) L.C. Chia & H.L. Fung	Z.Y. Cai & S.J. Zeng CZY104 (IBSC)
*Dendrocalamus* sp.	Z.Y. Cai & J.B. Ni CZY109 (IBSC)
*Gigantochloa brachystachya* Q.M. Qin, Y. Zeng & N.H. Xia	Y. Zeng 26 (IBSC)
*Gigantochloa maneensis* Q.M. Qin, N.H. Xia & J.B. Ni	Q.M. Qin et al. QQM319 (IBSC)
*Melocanna humilis* Kurz	H.L. Fung 780 (IBSC)
*Melocanna baccifera* (Roxb.) Kurz	H. Fung BG2711 (IBSC)
*Neololeba atra* (Lindl.) Widjaja	Z.Y. Cai CZY192 (IBSC)
*Neomicrocalamus prainii* (Gamble) Keng f.	C.J. Wang 1285 (SWFC)
*Neomicrocalamus* sp.	Z.Y. Cai & Z.Y. Niu CZY142 (IBSC)
*Temochloa* sp.	N.H. Xia et al. BH85 (IBSC)
*Schizostachyum dakrongense* N.H. Xia, Z.Y. Cai, Y.H. Tong & T.C. Vu	N.H. Xia et al. BVN20181114 (IBSC)
*Schizostachyum hainanense* Merr. ex McClure	Z. Huang 35281 (IBSC)
**Arundinarieae**	
*Acidosasa carinata* (W.T. Lin) D.Z. Li & Y.X. Zhang	Y.H. Tong et al. s. n. (IBSC)
*Acidosasa chinensis* C.D. Chu & C.S. Chao ex Keng f.	N.H. Xia et al. EHZ20190410 (IBSC)
*Acidosasa gracilis* W.T. Lin & X.B. Ye	Z.R. Zheng et al. ZXR210 (IBSC)
*Ampelocalamus actinotrichus* (Merr. & Chun) S.L. Chen, T.H. Wen & G.Y. Sheng	F.C. How 70138 (IBSC)
*Brachystachyum densiflorum* (Rendle) Keng	Y.T. Zhang s. n. (NF)
*Chimonobambusa marmorea* (Mitford) Makino	C.S. Chao 86017 (NF)
*Fargesia sapaensis* N.H. Xia & Y.Y. Zhang	N.H. Xia et al. 2018VNB-043 (IBSC)
*Fargesia spathacea* Franch.	P&a Investigation Team 0021 (NF)
*Ferrocalamus rimosivaginus* T.H. Wen	Sino-Soviet Botanical Expedition 2490 (PE)
*Ferrocalamus strictus* Hsueh & Keng f.	W.P. Zhang 840326 (SWFC)
*Gelidocalamus fengkaiensis* N.H. Xia & Z.Y. Cai	Z.Y. Cai CZY141 (IBSC)
*Indocalamus herklotsii* McClure	Y.H. Tong et al. BH259 (IBSC)
*Indocalamus longiauritus* H&.-Mazz.	Z.Y. Cai CZY55 (IBSC)
*Indocalamus sinicus* (Hance) Nakai	H.G. Ye 5416 (IBSC)
*Indosasa hispida* McClure	Z.R. Zheng et al. ZXR144 (IBSC)
*Indosasa shibataeoides* McClure	N.H. Xia et al. XNH40 (IBSC)
*Indosasa singulispicula* T.H. Wen	Z.Y. Niu NZY34 (IBSC)
*Khoonmengia honbaensis* N.H. Xia, Y.H. Tong & X.R. Zheng	N.H. Xia et al. BVN2017048 (IBSC)
*Menstruocalamus sichuanensis* (T.P. Yi) T.P. Yi	Y.T. Zhang s. n. (NF)
*Oligostachyum oedogonatum* (Z.P. Wang & G.H. Ye) Q.F. Zheng & K.F. Huang	N.H. Xia et al. XNH99 (IBSC)
*Oligostachyum shiuyingianum* (L.C. Chia & But) G.H. Ye & Z.P. Wang	Nan Zhu 2862 (IBSC)
*Oreocalamus Utilis* Keng	Y.C. Yang 3075 (IBSC)
*Petrocalamus luodianensis* (T.P. Yi & R.S. Wang) Z.P. Wang & W.Y. Zhang	Y.Y. Zhang s. n. (IBSC)
*Petrocalamus microphyllus* (Hsueh & T.P. Yi) Z.P. Wang & N.X. Ma	Z.Y. Cai & Y.T. Zhang CZY187 (IBSC)
*Phyllostachys bambusoides* Siebold & Zucc.	Z.R. Zheng et al. ZXR174 (IBSC)
*Phyllostachys danxiashanensis* N.H. Xia & X.R. Zheng	X.R. Zheng et al. ZXR196 (IBSC)
*Pleioblastus* × *kongosanensis* Makino	Z.Y. Cai CZY82 (IBSC)
*Pleioblastus* sp.	N.H. Xia et al. XNH166 (IBSC)
*Pseudosasa cantorii* (Munro) Keng f. ex S.L. Chen & et al.	N.H. Xia & Z.Y. Cai XNH186 (IBSC)
*Pseudosasa palidiflora* (McClure) S.L. Chen & G.Y. Sheng	W.T. Tsang 20216 (SYS)
*Ravenochloa wilsonii* (Rendle) D.Z. Li & Y.X. Zhang	T.P. Yi 108411 (IBSC)
*Sasamorpha sinica* (Keng) Koidz.	M.B. Deng 4149 (IBSC)
*Semiarundinaria fastuosa* (Mitford) Makino	M. Togasi 392 (PE)
*Shibataea* sp.	Z.Y. Cai & L. Xu CZY191 (IBSC)
*Sinosasa longiligulata* (McClure) N.H. Xia, Q.M. Qin & J.B. Ni	Q.M. Qin QQM182 (IBSC)
*Yushania rugosa* T.P. Yi aff.	Y.H. Tong & Z.Y. Cai TYH2130 (IBSC)
*Yushania* sp.	Z.Y. Cai & Z.X. Zhang CZY153 (IBSC)

## Data Availability

Data are contained within the article.
